# The Activation of the Tumor Suppressor Protein p53 by Acriflavine Leads to Mitochondrial Dysfunction and Improves the Radiosensitivity of Colon Cancer Cells

**DOI:** 10.1155/2022/1328542

**Published:** 2022-07-29

**Authors:** Caizhao Lin, Xiaohua Chen, Huayun Qiu, Benfeng Li, Min Guo

**Affiliations:** ^1^From the Department of Colorectal Surgery, The First Affiliated Hospital, College of Medicine, Zhejiang University, China; ^2^Department of Biochemistry, Medical College of Shaoguan University, Shaoguan, Guangdong Province 512026, China; ^3^Department of Nursing, Medical College of Shaoguan University, 108 Xinhua South Road, Wujiang District, Shaoguan, Guangdong 512026, China; ^4^Department of Oncology, The Fifth Affiliated Hospital, Southern Medical University, Guangzhou, Guangdong 510515, China

## Abstract

Colon cancer ranks third worldwide, and it has a growing incidence with urbanization and industrialization. Drug resistance in colon cancer is gradually affecting the treatment. This study focused on the mechanisms by which acriflavine (ACF) enhances the radiosensitivity of colon cancer cells. First, the expression and activation levels of tumor suppressor protein p53 were shown high in normal cells and tissues in its detection, which suggests that p53 is likely to be a key factor in colon cancer. Then, the expression of p53 ended up increasing in ACF group after SW620 cells were cultured with ACF. In addition, ACF group had some other changes. The expression of mitochondrial related antiapoptotic protein Bcl-2 increased, while the expression of proapoptotic protein Bax, Bad, cytopigment C, and apoptotic inducer AIF decreased. At the same time, the ability of apoptosis was enhanced, and the ability of proliferation and invasion was decreased. This suggests that ACF can promote p53 expression and affect mitochondrial function and the radiosensitivity of SW620. The luciferase reporting experiment showed that there was a binding site between ACF and p53. Besides, when IR treatment was applied to SW620 with high p53 expression, there was an increase in the expression of Bcl-2 in SW620 and decrease in Bax, Bad, and cytopigment C in AIF. Meanwhile, the cell apoptosis became stronger, and the proliferation and invasion became weaker. The experimental results were similar to those of SW620 cells cultured with ACF, suggesting that p53 is an intermediate factor in the regulation of SW620 by ACF. Finally, in this study, cells were cultured with ACF, and p53 was knocked down at the same time. The experimental results showed that after p53 was knocked down. ACF's ability to regulate SW620 is partially removed. This confirms the view that ACF regulates SW620 cells by regulating p53. In summary, this study found the mechanism by which ACF causes mitochondrial dysfunction and improves the radiosensitivity of colon cancer cells by activating the tumor suppressor protein p53, which may contribute to solving the drug resistance in colon cancer.

## 1. Introduction

Colon cancer ranked third worldwide [1], and it has a growing incidence with the development of urbanization and industrialization [2]. The one-year and five-year survival rates of colon cancer patients diagnosed from 1995 to 1999 were 72% and 54%, respectively [3]. At present, due to the development of technology and science, the survival rate of patients has improved, but the five-year survival rate is still less than 60%. The occurrence of colon cancer is usually random, the genetic colon cancer accounts for less than 5% [4], and diet is the most important factor leading to the occurrence of colon cancer. There are many treatments for colon cancer, but drug resistance is becoming a problem that cannot be ignored in the treatment of colon cancer. At the same time, adjuvant radiotherapy is also an effective means of treatment, and antiradiation cancer cells are still the main treatment obstacle of colon cancer [5]. Therefore, improving the radiotherapy sensitivity of cells can help improve the therapeutic effect of cancer.

Acriflavine (ACF) is a mixture of 3, 6-diamino-10-methyl acridine (trypaflavine) and 3, 6-diamino-acridine (proflavine), which has been used as a preservative and wound healing agent [6]. It has been revealed that ACF can inhibit many cancers, including osteosarcoma, breast cancer, brain cancer, lung cancer, liver cancer, and pancreatic cancer [7–11]. ACF can also act on AIDS patients as an antiviral drug without major side effects [12]. It has been confirmed by many studies that ACF can play a certain inhibitory role in colon cancer. Studies have shown that ACF action on colon cancer cells can activate endoplasmic reticulum stress and enhance cell apoptosis [13]. ACF plays a significant role in enhancing the sensitivity of drug-resistant cells and chemotherapy sensitivity of cancer cells [14, 15]. But it is still unclear whether ACF can improve the radiosensitivity of colon cancer in radiotherapy to improve the therapeutic effect.

p53, a tumor suppressor protein, regulates the cell cycle, DNA replication, and uncontrolled cell division during tumorigenesis [16]. When p53 protein aggregates or mutates and loses its original function, it will lead to the occurrence and progression of tumors. In addition, p53 can also play a direct role in mitochondria and participate in mitochondrial processes, leading to cell apoptosis [17]. Studies have shown that p53 mutations occur in more than half of cancers, suggesting that intact p53 plays an indispensable role in inhibiting tumor development [18]. And targeting p53 is a future direction of cancer treatment. However, whether p53 can regulate the radiosensitivity of colon cancer during chemotherapy and the mechanism between p53 and ACF is still unclear.

This study explored the mechanism by which ACF affects mitochondrial function of colon cancer cells and improves radiosensitivity by regulating p53. In the experiment, cancer tissues and cell lines SW620 of colon cancer patients were obtained, cells were treated with ACF and IR (ionizing radiation), and the expression level of p53 in colon cancer tissues and cells was detected, as well as the activity of cell physiological functions. This may provide new insights into how to tackle resistance in colon cancer.

## 2. Materials and Methods

### 2.1. Materials

The normal human colon epithelial cell line CCT-18co and colon cancer cell line SW620 were purchased from ATCC (American Type Culture Collection). 10% fetal bovine serum, 1% penicillin, and 1% streptomycin were added to Rpmi-1640 medium and then cultured with 5%CO2 at 37°C.

Colon cancer tissue and normal tissue adjacent to the cancer were taken from hospitals and were derived from patients with colon cancer who had not been treated with radiation. All patients obtained informed consent and signed informed consent forms. At the same time, this study has obtained the ethical approval of The First Affiliated Hospital, College of Medicine, Zhejiang University.

### 2.2. Quantitative Real-Time PCR

CCD-18co cells and SW620 cells were collected after treatment or culture, and 1 ml Trizol (Merck) was added to the cells, respectively, and lysed for 5 minutes. It was left on ice for 3 minutes, then mixed well with 0.2 ml chloroform addition. It was placed on ice for another 3 minutes, then centrifuged at 12000 rpm for 15 minutes at 4°C. The water phase was taken and left on ice for 10 minutes after adding 500 *μ*l isopropyl alcohol. It undertook centrifugation 12000 rpm for 10 minutes at 4°C. Centrifuge at 10000 rpm for 5 min at 4°C, then remove the supernatant. Finally, 20 *μ*l ddH2O was added, and the mixed RNA was blown repeatedly to obtain the RNA solution. The concentration and purity of RNA were determined by ultraviolet absorption method. 2.5 *μ*l of cdna, 5 *μ*l of SYBER Green (Takara), 0.5 *μ*l of upstream and downstream primers with a concentration of 30 *μ*M, and 1.5 *μ*l of DEPC water were used to form a 10 *μ*l system for PCR amplification. Record the data.

The primers used are as follows:

p53: forward: GAGGATTCACAGTCGGATA; reverse: ATCATCTGGAGGAAGAAGTT.

Bcl-2: forward: ATCCAGGACAACGGAGGCTG; reverse: CAGATAGGCACCCAGGGTGA.

Bax: forward: GATCGAGCAGGGCGAATG; reverse: CATCTCAGCTGCCACTCG.

Bad: forward: CTTGAGGAAGTCCGATCCCG; reverse: GCTCACTCGGCTCAAACTCT.

### 2.3. Western Blot

The CCD-18co cells and SW620 cells after treatment or culture digested with 0.25% trypsinase for 5 min and underwent centrifuge at 1000 rpm for 10 min. Then, 0.01 m concentration of PBS was used to wash them twice, and the cells were resuspended. Centrifugation at 13000 rpm for 30 min at 4°C, supernatant was taken. The 100 *μ*l supernatant was added to 10 *μ*l coomassie bright blue (Solarbio), the absorbance was measured by spectrophotometer, and the protein content was calculated by standard curve. 100 *μ*l supernatant with 20 *μ*l loading buffer was boiled at 100°C for 5 minutes to denature the protein. After it cooled down on ice for 3 min, the sample was obtained by centrifugation at 13000 rpm for 15 minutes at 4°C. SDS-PAGE gel was used for electrophoresis with concentrated gel running. A suitable PVDF membrane was pretreated with a transmembrane solution after being immersed in methanol for 2 minutes. The PVDF membrane and the PAGE gel after electrophoresis were assembled, and the membrane was transformed at 0°C and 70 V for 2 hours. 5% skimmed milk powder was sealed on a shaker for 1 hour and underwent incubation with primary antibody for 24 h at 4°C. The antibody used to detect the protein expression is as follows: mouse anti-AIF (1 : 800 dilution; Invitrogen), mouse anti-Bcl-2 (1 : 1000 dilution; Invitrogen), mouse anti-Bax (1 : 1000 dilution; Invitrogen), rabbit anti-Bad (1 : 1000 dilution; Invitrogen), mouse anti-p53 (1 : 1000 dilution; Invitrogen), and mouse anticytochrome C (1 : 1000 dilution; Invitrogen). The PBST was washed for 5 times, adding secondary antibody, and incubated for 2 hours. The PBST was washed with pure water for 5 times. The chemiluminescence system was used for 3 minutes, and the images were recorded.

### 2.4. MTT

Cell suspension was prepared as above. The concentration was adjusted to 50000/mL by cell counting. The planking was done using a 96-well plate with 100 *μ*l cell suspension per well, approximately 5000 cells. They were then incubated at 5%CO2 at 37°C for 24 hours. Add 10 *μ*l MTT (Procell, Wuhan) solution to each well and continue to culture for 4 hours. Discard the liquid from the well and terminate the culture. Lay several sheets of filter paper on a table and gently invert the 96-well plate to remove the supernatant. Add 100 *μ*l dimethyl sulfoxide to each well and shake on a shaker at low speed for 10 minutes to dissolve the Formazan crystals completely. Finally, the light absorption value of each well was measured at OD 490 nm of ELISA.

### 2.5. In Vitro Invasion Experiment

After the treated cells were dealt with the same in western blot, they were washed twice with 0.01 m concentration of PBS with discarding the culture medium and resuspended in serum-free medium containing BSA. The cell density was adjusted to 10000 cells/ml. Then, 100 *μ*l of cell suspension was inoculated into two Transwell chambers (invasion was measured with matrix gel, and migration was measured without matrix gel) and then cultured in an incubator for 24 h. Cells are removed from the orifice, the medium is aspirated, and the upper chamber is wiped with a cotton swab to remove the matrix glue and unmigrated cells. Take a new orifice, add 600 *μ*l 4% paraformaldehyde to it, place the chamber in the orifice and fix it for 20 minutes. Discard the fixation solution, stain with 0.1% crystal violet for 10 min, wash with 0.01 m concentration OF PBS for 3 times, remove the crystal violet that is not bound to the cells, gently wipe the upper side of the chamber with a cotton swab, and erase the dye that is not specifically bound to the upper surface of the chamber for subsequent microscopic examination. After proper air drying, the cells were observed and counted in 5 fields under a high power microscope.

### 2.6. Flow Cytometry

Cells in logarithmic growth phase underwent the same procedure as above, and the culture medium was discarded. PBS was removed by centrifugation, and 70% ethanol precooled with ice was added and fixed at 4°C for 2 hours. The fixation solution was discarded by centrifugation and resuspended in 0.01 m PBS for 5 min. PBS was discarded. Dye with 1 ml PI dye solution (Merck) for 30 min at 4°C to avoid light. Upflow cytometry detection: the fluorescence of PI was excited by argon ion, the wavelength of laser light was 488 nm, the wavelength of emitted light was greater than 630 nm, and the red fluorescence was generated. The histogram of PI fluorescence intensity could also be used to analyze the scatter diagram of the scattered light on the opposite side of the prescattered light.

### 2.7. Immunohistochemical Detection

The obtained colon cancer tissue and adjacent normal tissue were fixed with 4% paraformaldehyde, embedded in paraffin, and sectioned with a thickness of 5 *μ*m. The tissue sections were baked at 60°C for one hour until the wax melted. The slices were dewaxed in xylene for 15 minutes. They were soaked with 95%, 90%, 80%, and 70% ethanol for 2 minutes, respectively, after immersed in 100% ethanol for 5 minutes, and then, 0.01 m PBS was used to wash for 3 times. Antigenic repair was completed when the tissue sections were placed in a pressure cooker with a citrate buffer and boiled for 5 minutes. Then, distilled water and 0.01 m PBS were used to wash twice. Specific primary antibody was added after 5% bovine serum protein was sealed for 10 min and incubated at 4°C overnight. It was washed with 0.01 m PBS for 3 times. Then, secondary antibody drops were dropped and incubated at 37°C for 2 hours and repeated the washing procedure. After DAB color developing for 5 minutes, its floating color was washed off with distilled water to wash off, and it was redyed hematoxylin for 10 seconds. The sections were dehydrated with gradient ethanol for 2 minutes at each concentration, and the xylene was transparent for 5 minutes. Resin was employed to seal the film, and we observed under microscope and photographed.

### 2.8. Statistics and Analysis

The significance of the data in the experiment was evaluated by univariate variance and T-text.

## 3. Results

### 3.1. The Expression Level of p53 in Normal Tissues and Cells Is Higher than That in Colon Cancer Cells and Tissues

**Figure 1:**
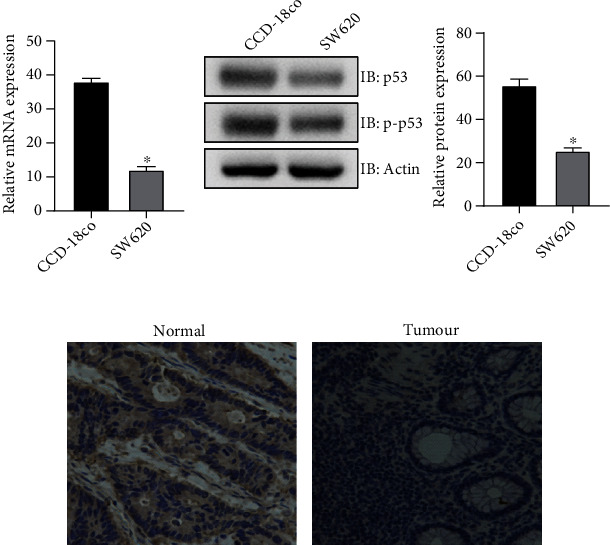
Low expression of p53 in colon cancer tissues and cells. (a) p53 expression was low in colon cancer cells by QRT-PCR. (b) Low expression of p53 in colon cancer cells by western blot. (c) Low expression of p53 in colon cancer tissues. ∗*P* < 0.15, compare with the CCD-18co group.

The purchased normal human colon epithelial cell line CCD-18co and colon cancer cell line SW620 were primary cultured and passed. The results showed that the expression of p53 mRNA in CCD-18co was higher than that in SW620 (Figure 1(a)), and it is true for its protein expression level (Figure 1(b)). The protein expression of p53 in colon cancer tissues was low, while that in normal adjacent tissue was high (Figure 1(c)). The above results showed that there was a lower p53 expression in colon cancer cells and tissues, suggesting that p53 may be a key factor in the treatment of colon cancer.

### 3.2. ACF Promotes the Expression and Activation of p53

In radiotherapy, ACF will cause mitochondrial dysfunction and enhanced radiosensitivity of SW620 cells.

**Figure 2:**
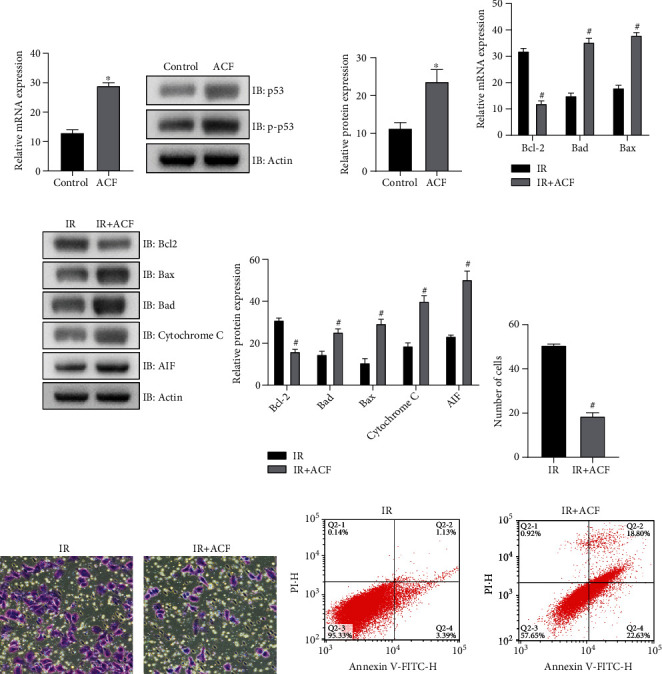
ACF promotes the expression and activation of p53, affects the mitochondrial function of colon cancer cells, and enhances radiosensitivity. (a) The expression of p53 mRNA was increased after ACF treatment. (b) The expression level of p53 became higher after ACF treatment. (c) After ACF treatment, there was a lower the mRNA expression of Bcl-2 and higher mRNA expression levels of Bad and Bax. (d) After ACF treatment, the expression of Bcl-2 decreased, and the expressions of Bad, Bax, AIF, and cytochrome C increased. (e) The proliferation ability of SW620 cells was weakened. (f) The invasion ability of SW620 cells was inhibited. (g) Apoptosis of SW620 cells was enhanced. ∗*P* < 0.15, compare with the control group. ^#^*P* < 0.15, compare with the IR group.

Cultured SW620 cells were taken and treated with ACF. After 7 days of culture, the expression level and phosphorylation level of p53 were detected. The mRNA expression of p53 increased after ACF treatment in comparison with the control group (Figure 2(a)). The protein expression and phosphorylation level of p53 in ACF group were increased to a certain extent (Figure 2(b)). These results suggest that ACF treatment of SW620 can promote the generation and activation of p53. Then, the cells were cultured with ACF and treated with IR (ionizing radiation). One week later, some changes took place when compared with the IR group. There was a decrease in the mRNA expression of antiapoptotic protein Bcl-2 and increase in the mRNA expression of proapoptotic proteins Bad and Bax (Figure 2(c)). The protein expression of Bcl-2 became lower, while the expressions of Bad, Bax, apoptosis-inducing factor AIF, and cytochrome-C were enhanced (Figure 2(d)). These results showed that simultaneous treatment of ACF and IR resulted in mitochondrial dysfunction and enhanced apoptosis in SW620 cells. The proliferation of SW620 cells was reduced (Figure 2(e)). In vitro invasion assay showed that the invasion of SW620 cells was inhibited (Figure 2(f)). Flow cytometry showed that SW620 cells were more apoptotic (Figure 2(g)). The above results demonstrated that viability of SW620 cells treated with IR and ACF decreased compared with SW620 cells treated with IR alone, while ACF increased the radiosensitivity of SW620 cells.

### 3.3. The High Expression of p53 Resulted in Mitochondrial Dysfunction and Increased Radiosensitivity of SW620 Cells during IR Process

**Figure 3:**
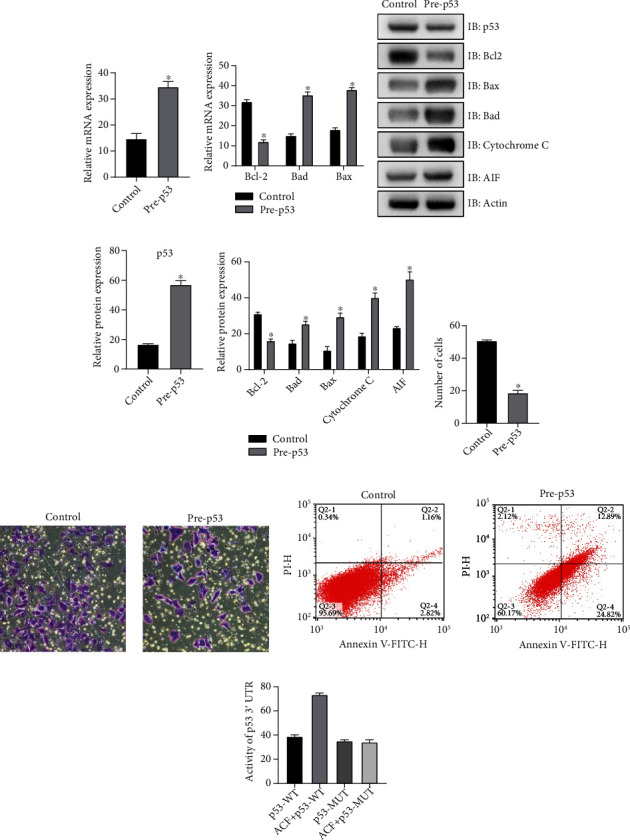
Overexpression of p53, mitochondrial dysfunction, and enhanced radiosensitivity of SW620 cells. (a) Overexpression of p53 was detected successfully by QRT-PCR. (b) The experimental results of QRT-PCR showed that the mRNA expression levels of Bcl-2 decreased with overexpression of p53, while the mRNA expression levels of Bad and Bax increased. (c, d) Western Blot results showed that overexpression of p53 led to a decrease in the expression level of Bcl-2, while the expressions of Bad, Bax, AIF, and cytochrome C increased. (e) MTT results showed that overexpression of p53 weakened the proliferation ability of SW620 cells. (f) The results of in vitro invasion experiment showed that the invasion ability of SW620 cells was inhibited by overexpression of p53. (g) Flow cytometry showed that apoptosis of SW620 cells was enhanced. (h) Luciferase reporting experiment results showed that there was a binding site between p53 and ACF. ∗*P* < 0.15, compare with the control group.

Cultured SW620 cells were taken, and p53 was overexpressed and transferred into cells. The experiment showed the expression of p53 mRNA was increased (Figure 3(a)). These results show that overexpression is effective. Then, SW620 cells were treated with IR for one week, and we got the following results. The mRNA expression level of Bcl-2 was lower in the overexpressed P52 group, while the mRNA expression level of Bad and Bax was increased (Figure 3(b)). The expression of p53 protein increased, and the protein expression of Bcl-2 was less in the overexpression group, while there was an increase in the expressions of Bad, Bax, apoptosis-inducing factor AIF, and cytochrome C (Figures 3(c), and 3(d)). The proliferation, invasion, and apoptosis of SW620 were detected by MTT, invasion test in vitro, and flow cytometry. The experimental results showed that the overexpression of p53 reduced the proliferation and invasion of SW620 cells during the IR process and enhanced the apoptotic ability (Figures 3(e)–3(g)). The results of this study were similar to the experimental results of ACF treatment of SW620 cells, and ACF can promote the production and activation of p53, suggesting that ACF may affect SW620 cells by regulating p53. The luciferase reporting experiment showed a binding site between p53 and ACF (Figure 3(h)), which further supported the view that p53 was the intermediate factor of ACF in regulating SW620.

### 3.4. Inhibition of p53 Expression Can Eliminate the Regulatory Effect of ACF on SW620 Cells

**Figure 4:**
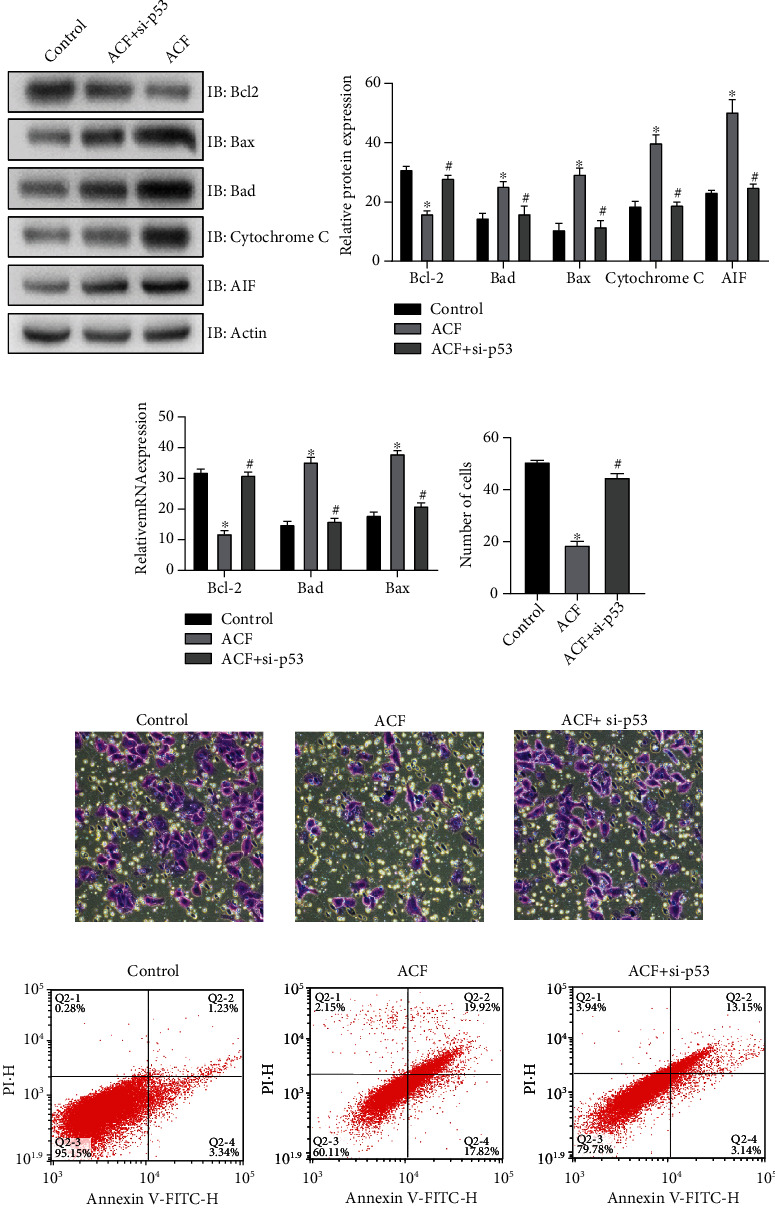
Inhibition of p53 expression, the regulatory effect of ACF on SW620 cells was partially relieved. (a) Western blot results showed that after p53 deletion, the expression level of Bcl-2 increased, while the expressions of Bad, Bax, AIF, and cytochrome C decreased. (b) The experimental results of QRT-PCR showed that the regulation of Bcl-2, Bad, Bax, AIF, and cytochrome C was partially relieved by ACF after p53 was knocked down. (c) MTT test results showed that cell proliferation increased after p53 deletion. (d) The results of in vitro invasion experiment showed that the invasion ability of cells recovered after p53 deletion. (e) Flow cytometry results showed that the apoptosis ability of cells was weakened after p53 deletion. ∗*P* < 0.15, compare with the control group. ^#^*P* < 0.15, compare with the ACF + si − p53 group.

The cultured SW620 cells were classified into 3 groups, the control group, the group cultured with ACF, and the group was cultured with ACF after knocking down the expression of p53. Finally, the cells were treated with IR for one week, the experiments showed that after p53 deletion, the expression level of Bcl-2 was higher, but still lower than that of the control group, while the expressions of Bad, Bax, AIF, and cytopigment C were decreased, but still higher than that of the control group (Figure 4(a)). The results of QRT-PCR were similar to those of western blot (Figure 4(b)). The results of MTT detection of cell proliferation showed that SW620 cells cultured with ACF alone had the weakest tissue capacity. After p53 deletion, the proliferation capacity was improved but still lower than that of the control group (Figure 4(c)). The invasion ability of SW620 cells in the p53 knockdown group was weaker compared with the control group, but stronger in contrast with ACF group (Figure 4(d)). Flow cytometry showed that SW620 cells in ACF group had the strongest apoptotic ability, followed by p53 knockdown group, and the weakest apoptotic ability in control group (Figure 4(e)). The above experimental results indicate that, after p53 knockdown, the regulatory ability of ACF on SW620 cells in IR process is partially removed. It is proved that ACF can affect the mitochondrial function and radiosensitivity of SW620 cells by regulating p53.

## 4. Discussion

Colon cancer is a fairly common cancer. In the United States, about 150,000 people are diagnosed with colon and rectal cancer each year, and 50,000 of them die. In Europe, about 250,000 people are infected each year, and globally, the figure is 1 million [19–21]. Patients with colon cancer may face considerable problems, such as abdominal pain, blood in the stool, black stool, weakness, and changes in bowel habits [22, 23]. These side effects can greatly affect the quality of life of patients. Besides, drug resistance has gradually become an urgent problem in the treatment of colon cancer.

In this study, we focused on the mechanism by which ACF improves the radiosensitivity of colon cancer cells during radiotherapy and thus improves the therapeutic effect. ACF was one of the first antimicrobials and preservatives to be discovered, but it has found many new uses. High-throughput molecular screening revealed that ACF is an antitumor agent in colon cancer [24]. Some studies have shown that ACF can enhance the toxicity of chemotherapeutic compounds to cancer cells [25, 26]. ACF is also commonly used as an inhibitor of HIF protein expression, which promotes the progression of various solid tumors and leukemia [27].

Mitochondrial dysfunction is an important marker of cancer. The tumor suppressor p53 regulates mitochondrial respiration and cell metabolism [28]. Other studies have shown that p53 can interact with members of the Bcl-2 protein family to induce mitochondrial membrane permeabilization and promote cell apoptosis [29]. Cytochrome C and AIF, as mitochondrial apoptosis factors, are regulated by bcl-2 protein family and control cell apoptosis [30]. This study focused on the regulatory relationship between ACF and tumor suppressor protein p53 and its effect on the radiosensitivity of colon cancer cells.

First, colon cancer cell SW620 and normal human colon epithelial cell line CCD-18co, as well as colon cancer tissue and adjacent normal tissue, were obtained in this study. p53 expression detected showed higher in normal cells and tissues than in colon cancer counterparts. The difference of p53 expression suggests that p53 may play an inhibitory role in the development of colon cancer.

Subsequently, colon cancer cells were treated with ACF and IR. We found that ACF can promote the expression and activation of p53 and increased the radiosensitivity of SW620 cells. The expression levels of Bcl-2 decreased, while the expression levels of Bax, Bad, cytochrome C, and AIF increased. Mitochondrial death was affected by the proportion of antiapoptotic protein Bcl-2 and proapoptotic electrowhite Bax and Bad. When apoptotic signal is generated, Bax will migrate from cytoplasm to mitochondrial outer membrane, causing mitochondrial dysfunction, stomata, release cytopigment C, and apoptosis-inducing factor AIF into cytoplasm, and then induce apoptosis [31]. Combined with the results of this study, ACF culture of SW620 cells resulted in mitochondrial dysfunction and promoted apoptosis.

Then, p53 was overexpressed in this study, and the cells were also treated with IR. We got the following results. After the overexpression of p53, the expression level of Bcl-2 decreased, and the expression level of Bax, Bad, cytochrome C, and AIF increased. The proliferation and invasion of SW620 cells were decreased, while the apoptosis was enhanced. This is similar to the experimental results of ACF treatment of SW620 cells. The luciferase reporting assay showed that there was a binding site between ACF and p53. These results suggest that ACF may affect mitochondrial function and radiosensitivity of SW620 cells by promoting the expression and activation of p53.

Finally, in order to confirm that p53 is an intermediate factor of ACF regulating SW620 cells, the expression of p53 was first knocked down in this study, and then ACF culture and IR treatment were used. The experimental results showed that after p53 knockdown, the expression of Bcl-2 increased, while the expression of Bax, Bad, cytochrome C, and AIF decreased. The proliferation and invasion of SW620 cells were increased, but still lower than that of the control group. The apoptotic ability is reduced. This indicates that the regulatory effect of ACF on SW620 cells is partially weakened after the inhibition of p53, which supports the view that ACF regulates SW620 cells through p53.

## 5. Conclusion

In summary, the present study found that ACF can affect the mitochondrial function of colon cancer cells and improve their radiosensitivity during radiation therapy by promoting the generation and activation of p53, which contributes to solving the drug resistance in colon cancer treatment.

## Data Availability

All the data during the current study are included in the article.
